# Experimental Study on Electrical Conductivity of Several [C4mim][BF4] Ionic-Liquid-Based Nanocolloids

**DOI:** 10.3390/nano13071224

**Published:** 2023-03-30

**Authors:** E. I. Chereches, A. A. Minea

**Affiliations:** Faculty of Materials Science and Engineering, Technical University “Gh. Asachi” of Iasi, Bd. D. Mangeron No. 63, 700050 Iasi, Romania

**Keywords:** electrical conductivity, nanocolloids, ionic liquid, experimental

## Abstract

Nanocolloids are receiving considerable attention in regard to their properties and future applications, especially as heat transfer fluids and phase change materials for energy storage. Additionally, studies on ionic liquids and ionic-liquid-based nanocolloids are at the forefront of research preoccupations. This study aims to shed light on applications of nanocolloids based on [C4mim][BF4] ionic liquid, giving insight into the electrical conductivity of [C4mim][BF4] ionic liquid, as well as three types of nanoparticles suspended in this particular ionic liquid, namely Al_2_O_3_ (alumina), ZnO (zinc oxide) and MWCNT (multi-walled carbon nanotubes). In this experimental research, three types of suspensions were carefully prepared and the electrical conductivity was measured both at ambient temperature and during heating. The results are discussed in the context of the state of the art. The electrical conductivity variation with temperature was found to be linear, and nanoparticle loading significantly influenced the electrical conductivity of the suspensions. A complex analysis in terms of temperature and nanoparticle type and loading was performed. In conclusion, the electrical properties are relevant for many applications and further experimental work needs to be devoted to their study.

## 1. Introduction

In recent years, much research has been directed to the formulation of new fluids for a number of applications in electronics, heat transfer, chemical engineering and many other sectors. Nanoparticle-enhanced fluids are a particular case, and research is dedicated both to defining new fluids and measuring their properties, as well as to their application areas. Nevertheless, despite intense research, there are still points to be further discussed and clarified, and most concern fluids’ properties and their dependence on base fluid (BF) type, nanoparticle type, dimensions, concentrations and synergy between the base fluid and the nanoparticle (NP) type, as well as the electrical behavior, a less studied topic [1,2,3,4,5,6,7]. Discussions of electrical conductivity are connected to the capacity of charged NPs in the BF to carry the charges in the direction of corresponding electrodes once an electric potential is employed, as was affirmed by Sundar et al. [8] and Chereches and Minea [9]. Furthermore, electrical conductivity studies can complement the nanofluid stability studies, which can offer added value in describing these new fluids’ behaviors (see [10,11,12,13] for details).

Zyla and Fal [13] studied silicon dioxide–glycol nanofluids’ electrical conductivity and noticed a linear increase in electrical conductivity when the concentration of nanoparticles increased. The same linear increase in electrical conductivity was noticed for most of the nanofluids in the literature [14,15,16,17,18,19,20,21,22]. Furthermore, a detailed discussion in terms of the state of the art is given.

Banisi et al. [23] gathered relevant data on electrical conductivity models and highlighted their particular applications, which is helpful to gain some insights into the electrical conductivity of dispersions.

Even if studies on electrical conductivity of nanofluids with alumina nanoparticles are abundant (see [3,5,6]), ZnO and MWCNTs have been seldom considered. For example, several ZnO nanoparticle-enhanced fluids were studied by Shen et al. [21], who concluded that the electrical conductivity is influenced by Brownian motion and the accumulation of NPs, and thus the stability of the NF can also be estimated by electrical conductivity means.

Caglar et al. [12] discussed the electrical conductivity of ZnO nanostructured materials and concluded that the electrical conductivity of ZnO is thermally triggered, while Sharma et al. [14] highlighted ZnO applications in optoelectronics, field effect transistors, solar cells, photoluminescence devices and diluted magnetic semiconductors.

On the other hand, Shoghl et al. [22] performed a complex experimental work employing CuO, TiO_2_, MgO, MWCNT, Al_2_O_3_ and ZnO nanoparticles and demonstrated that all the studied NFs have increased electrical conductivity in comparison with the corresponding BFs.

White et al. [24] investigated the electrical conductivity of ZnO–propylene glycol nanofluids and noticed a rise in electrical conductivity with NP loading and decreasing NP size. Moreover, from the authors’ discussion of the results, it was noticed that the electrical conductivities of NFs drop meaningfully below the linear fit that has been demonstrated by other authors.

MWCNT-based nanofluids have been extensively investigated by many research groups, mostly in terms of thermal conductivity’s clear benefits. Nevertheless, electrical conductivity was not considered relevant and few studies are identified on this topic (see [4,11,15]). Giwa et al. [25] measured the electrical conductivity of deionized water with an MWCNT hybrid and demonstrated that the increase in temperature and concentration determines a rise in electrical conductivity. Glory et al. [4] studied MWCNT–water electrical conductivity and noticed that the electrical properties do not have a similar tendency to the thermal conductivity. The authors found that the electrical conductivity is mostly constant and decreases when the NP loading reaches 0.1 wt%; this phenomenon might be due to MWCNTs’ influence on the base fluid. In particular, the NPs might percolate inside the water chemical structure at low concentration values.

Bhattacharya et al. [26] prepared ethyl ammonium-nitrate-based ferrofluids with citrate-coated nanoparticles and sodium counterions in concentrations of up to 16%, and the thermoelectric potential was determined. The authors concluded that the inferred sign of the effective charge from the thermoelectric measurements is positive, while the Seebeck coefficient and the power output diminish with the addition of nanoparticles.

Bakthavatchalam et al. [27] prepared nanocolloids based on 1-ethyl-3-methyl imidazolium octyl sulfate ionic liquid and measured their electrical conductivity, which was found to increase with the addition of MXene NPs, reaching 571 μS/cm.

Based on the studies that consider base fluids, most of the investigated fluids are water, ethylene glycol, other glycols and several oils. The idea of involving ionic liquids in heat transfer applications is novel, and studies on nanoparticle-enhanced ionic liquids are scarce. Furthermore, no relevant conclusion was drawn from the limited studies that were identified in the most common databases, as well as the ionic liquids database [28].

Alizadeh et al. [29] added graphene nanoplatelets to [BMIM][PF6] and measured electrical conductivity at ambient temperature, noticing an increase with the rise in temperature and concentration. This group developed a systematic study on ionic liquids and nanoparticle-enhanced ionic liquids (see Chereches et al. [30,31]) and concluded that the electrical conductivity increases depending on the NP type, temperature and base fluid. Overall, research on the variation in electrical conductivity in nanofluids is still scarce; however, most of the studies highlight a dependence on nanoparticle type and concentration, and all experimental investigations on variations in electrical conductivity with temperature demonstrated a linear increase when temperature rises.

The main aim of this research was to investigate the electrical conductivity of several suspensions based on 1-butyl-3-methylimidazolium tetrafluoroborate ionic liquid enhanced with three different kinds of nanoparticles (i.e., Al_2_O_3_, ZnO and MWCNTs) with different electrical properties. We considered the evaluation of both pH and electrical conductivity, and some insight into nanocolloids’ stability is provided, building on the state of the art. The experimental study presented here can be seen as a continuation of our research group on the electrical conductivity of nanofluids involving different base fluids (in this study, the base fluid is an ionic liquid) and nanoparticle types, aiming to a shed some light on this understudied property.

## 2. Theoretical Models versus Experimental Ones

The electrical conductivity of nanocolloid suspensions is the least-studied property in regard to the implementation of new fluids. Nevertheless, several studies have acknowledged that theoretical models are not suitable, in most cases, to predict the electrical conductivity variation with the addition of nanoparticles. A review published on electrical conductivity was performed by Banisi et al. [23], where several models were acknowledged and four main categories were identified as classical solutions, ordered arrangements of dispersed phase, approximations involving no empirical parameters and relations involving empirical parameters [23]. Nevertheless, electrical conductivity is widely calculated with the help of Maxwell and Bruggeman equations [19,32], as can be inferred from the literature (see, for example [1,2,3,4,5,6,7,8,9,10,11,12,13,14,15,16,17,18,19,20,21,22,23]). 

In Table 1 are presented a few of the models available in the literature for electrical conductivity estimation.

## 3. Experimental Section

The chemicals (nanoparticles) used in this study were acquired from Sigma-Aldrich (St. Louis, USA), and their properties are listed in Table 2. The ionic liquid was from IoLiTec (Germany) and has the following properties: CAS no. 174501-65-6, Code IL–0012-HP, purity ≥ 99%, formula C8H15BF4N2, molecular weight of 226.02 g/mol, liquid state at 298.15 K, yellow to orange color, melting/freezing point of 190–198 K, glass transition temperature of 176 K, density (at 298.15 K) of 1.203 g/cm3, viscosity (at 298.15 K) of 103 mPa s, specific heat (at 298.15 K) of 351.5–364.8 J/(mol K), electrical conductivity (at 298.15 K) of 3.15 mS/cm, water content (KF) of 238 ppm. The ionic liquid, nanoparticles and suspensions were studied in terms of SEM, TEM, X-ray and porosity; further insights can be found in Chereches et al. [33,34,35].

The suspensions were prepared using a two-step method by suspending nanoparticles in the ionic liquid. The ratio between nanoparticles and ionic liquid quantity was first calculated to obtain the desired suspensions’ mass concentrations. An ENTRIS224l-1S balance from Sartorius AG (Goettingen, Germany) with 0.1 mg accuracy was employed for weighing all the chemicals. The substances were shifted into a 50 mL culture tube and sonicated for 60 min in a Geti GUC02A ultrasonic bath (with an ultrasound power of 60 W) at a frequency of 40 Hz. The entire preparation procedure was carried out at room temperature (i.e., 293.15 K) in static conditions.

Electrical conductivity was investigated by Edge^®^ Multiparameter HI 2030 (Hanna Instruments) equipment, with an integrated temperature sensor and large measurement areas, up to 500 mS/cm and 0.01 μS/cm resolution. The temperature measurement accuracy is ±0.2 K. The experiment was performed at ambient temperature, followed by heating up to 333.15 K employing a heating bath, and the data were recorded from 5 to 5 degrees. The testing accuracy was calculated as 3%. Prior to the tests, the equipment was calibrated with HI7031 solution (1413 μS/cm at 298.15 K). The entire experiment was carefully conducted and the electrode was cleaned with distilled water and dried after each measurement. Every test was repeated 3–5 times to minimize the experimental errors, and the recorded figures were calculated as the average value.

## 4. Results and Discussion

The experimental outcomes on electrical conductivity are discussed for all three classes of ionanofluids, as well as for the ionic liquid. The electrical conductivity of nanoparticles is described in Table 3.

Alumina nanoparticles are considered insulators with low electrical conductivity. Zinc oxide is one of the most relevant group II–VI semiconductor materials, having a wide bandgap with a direct energy gap of approximately 3.3 eV [12]. On the other hand, MWCNTs exhibit some of the highest electrical conductivities.

In this work, the Cruz model [10] was applied for each particular case, as is outlined in Table 3. 

### 4.1. Ambient Temperature Tests

**Figure 1:**
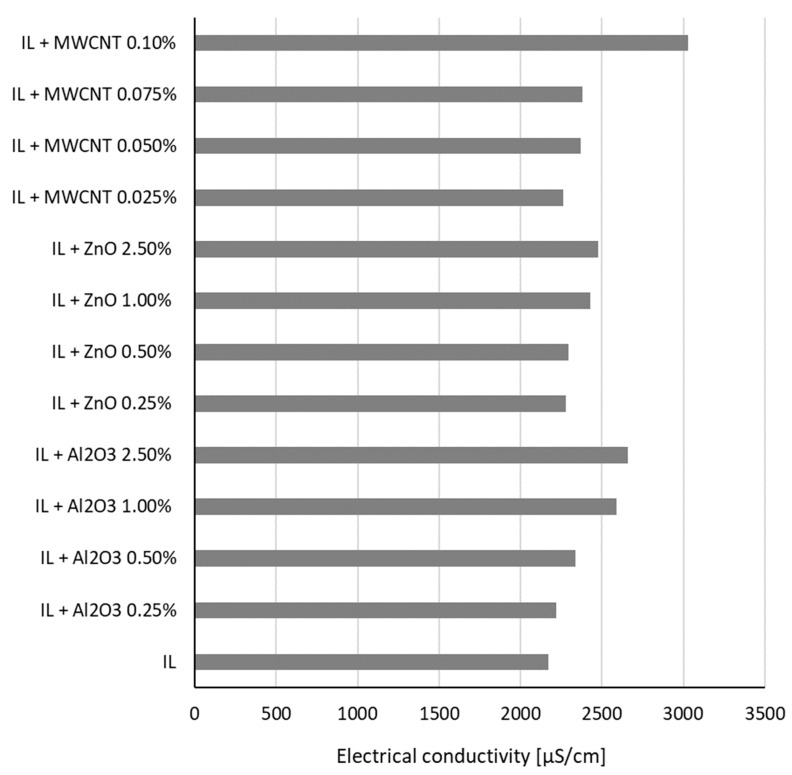
Experimental data of all samples at ambient temperature.

The experimental data of all the samples’ behavior at ambient temperature (i.e., 278.15 K) are given in Figure 1 for oxide suspensions and MWCNT suspensions.

From Figure 1, it can be seen that all suspensions have a higher electrical conductivity than the host fluid (i.e., ionic liquid, IL). The increase depends on the nanoparticle type and concentration, as is detailed here.

**Figure 2:**
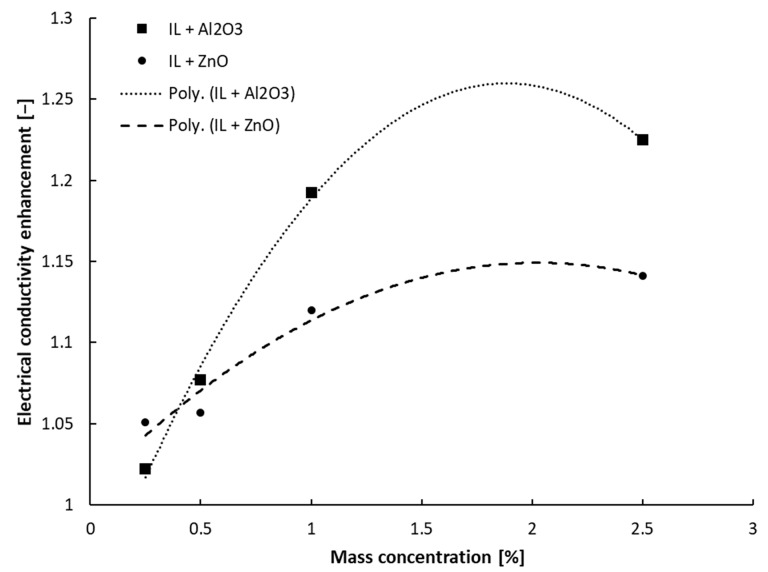
Electrical conductivity enhancement data of oxide samples at ambient temperature.

**Figure 3:**
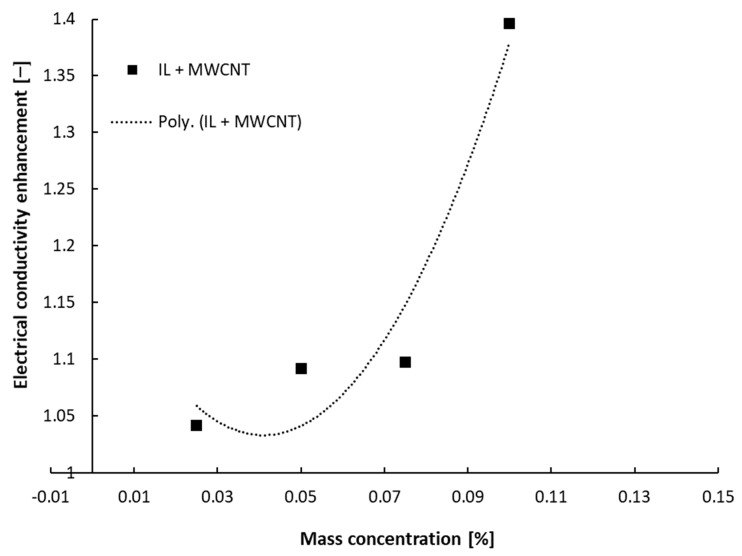
Electrical conductivity enhancement data of MWCNT samples at ambient temperature.

In Figure 2 and Figure 3, the results on electrical conductivity enhancement over the IL are summarized, and it can be noticed that the relative electrical conductivity dependence on nanoparticle concentration is a second-order polynomial one and not linear, as was defined in the open literature, e.g., for nanofluids with water or ethylene glycol (see [3] for in-depth discussion). The relative electrical conductivity (also called electrical conductivity enhancement) is defined as the ratio between the electrical conductivity of the suspension and that of the ionic liquid.

κ_r_ = κ_nf_/κ_f_ (1)

Several basic equations can be established as follows:

-For suspensions with alumina:

κ_r_ = −0.091 ϕ^2^ + 0.3428 ϕ + 0.937, R² = 0.9957 (2)

-For suspensions with ZnO:

κ_r_ = −0.0339 ϕ^2^ + 0.1373 ϕ + 1.0103, R² = 0.9519 (3)

-For suspensions with MWCNT:

κ_r_ = 99.539 ϕ^2^ − 8.1659 ϕ + 1.2007, R² = 0.9277 (4)

where κ_r_ is the relative electrical conductivity, ϕ is the mass concentration and R^2^ is the R-squared value calculated for each situation.

Referring to Figure 2 and Figure 3, it can be seen that the relative electrical conductivity increased to 1.4 for the ionanofluids, and higher values were attained when MWCNTs were inserted in the base ionic liquid. The electrical conductivity increased by 23% and 14% for samples with Al_2_O_3_ and ZnO, respectively.

The electrical conductivity increase is influenced by the electrical double layer (EDL) or the stability of the suspension. When NPs are hosted in the ionic liquid, the EDL is likely to appear and the nanoparticles become charged and might transfer this charge to the suspension, thus increasing the electrical conductivity of the ionanofluid. So, as the NP loading increases (i.e., the number of nanoparticles increases in the sample), the ionanofluid’s effective electrical conductivity rises. The authors’ explanation of this phenomenon is that the augmentation of the ionanofluids’ electrical conductivity is due to both EDL formation around each NP surface and the ionic structure of the base liquid (i.e., 1-butyl-3-methylimidazolium tetrafluoroborate), which has several nanoparticles. Furthermore, the results outlined in Figure 3 suggest the existence of a percolation threshold. Nevertheless, the proposed equations (i.e., Equations (2)–(4)) are subject to further experimental work on suspensions with different mass concentrations in order to clarify the aspect defined by a second-order polynomial variation. The higher nanoparticle concentration (i.e., 2.5 wt%) was chosen to determine what happens at these high amounts of nanoparticle loading, even if the highly concentrated suspensions are not performing well in real-world applications due to increased viscosity issues.

A similar observation was noticed by other research groups (see, for example [5,6,10,14]). In particular, Sarojini et al. [16] studied suspensions with Al_2_O_3_ and concluded that the electrical conductivity growth can be due to the formation of surface charges by the effect of NP polarization in a polar liquid (i.e., water). Ganguly et al. [5] considered that the occurrence of uniformly dispersed nanoparticles determines a high electrophoretic mobility, which increases the electrical conductivity, despite the character of the nanoparticles (i.e., even if the NPs are insulators, such as alumina). A similar observation can also be applied for this study, where the experiment revealed a higher electrical conductivity of suspensions with alumina compared to similar ZnO nanoparticle loadings. On the other hand, a small addition of MWCNTs (up to 0.1 wt%), which are conductors, determines an increase of 40% in electrical conductivity.

If the nanoparticle loading increases, the availability of conduction paths is higher in the sample, which improves the electrical conductivity. Concluding, Ganguly et al. [5] posited that the higher the electrical conductivity, the greater the nanofluid stability.

### 4.2. Tests with Temperature Variation

**Figure 4:**
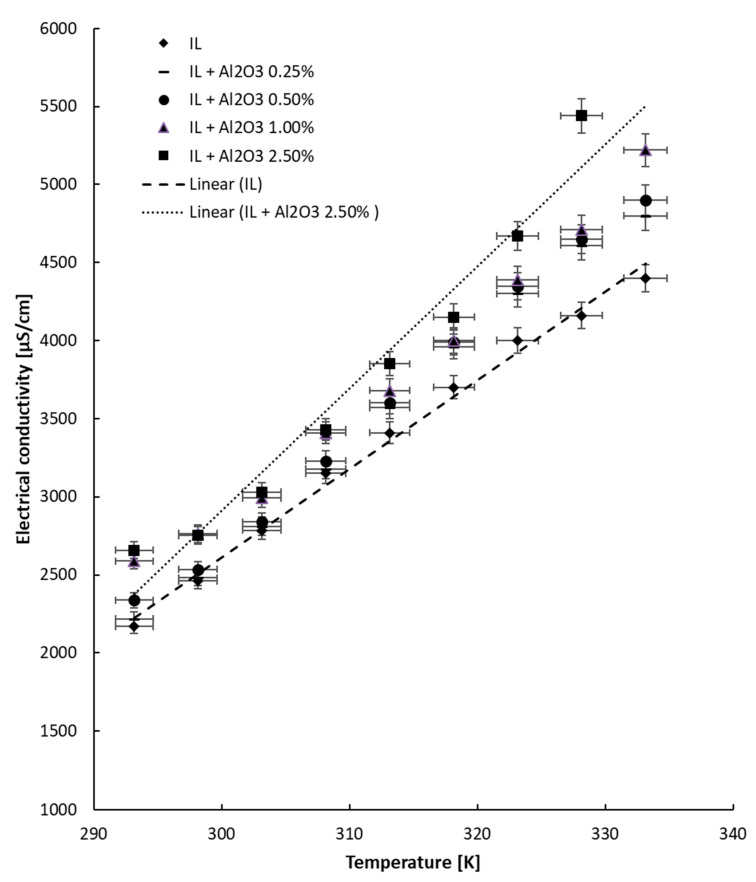
Experimental results for electrical conductivity variation with temperature: IL and Al_2_O_3_ + IL samples.

**Figure 5:**
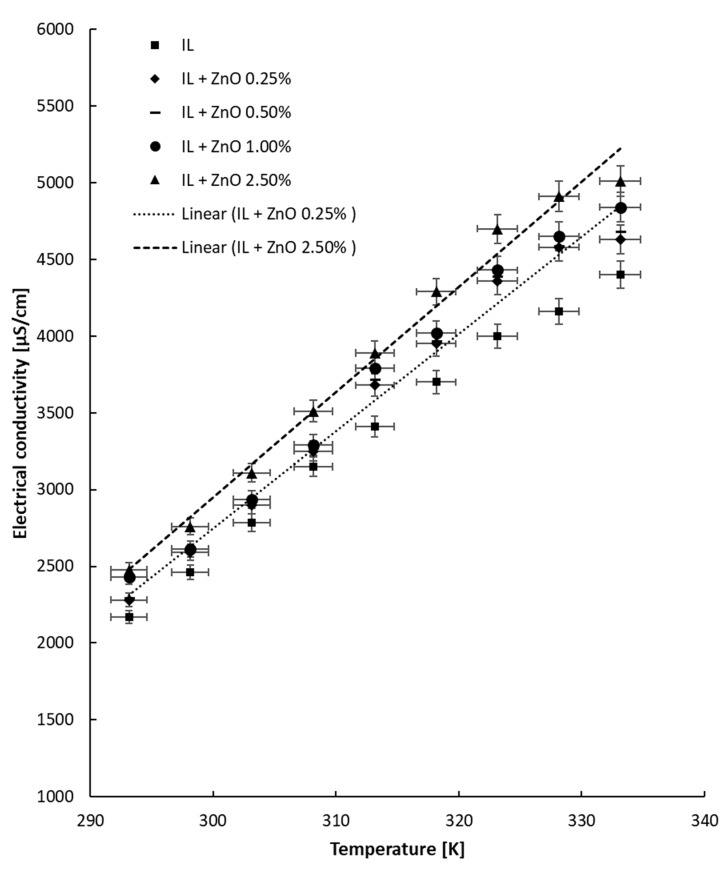
Experimental results for electrical conductivity variation with temperature: ZnO + IL samples.

**Figure 6:**
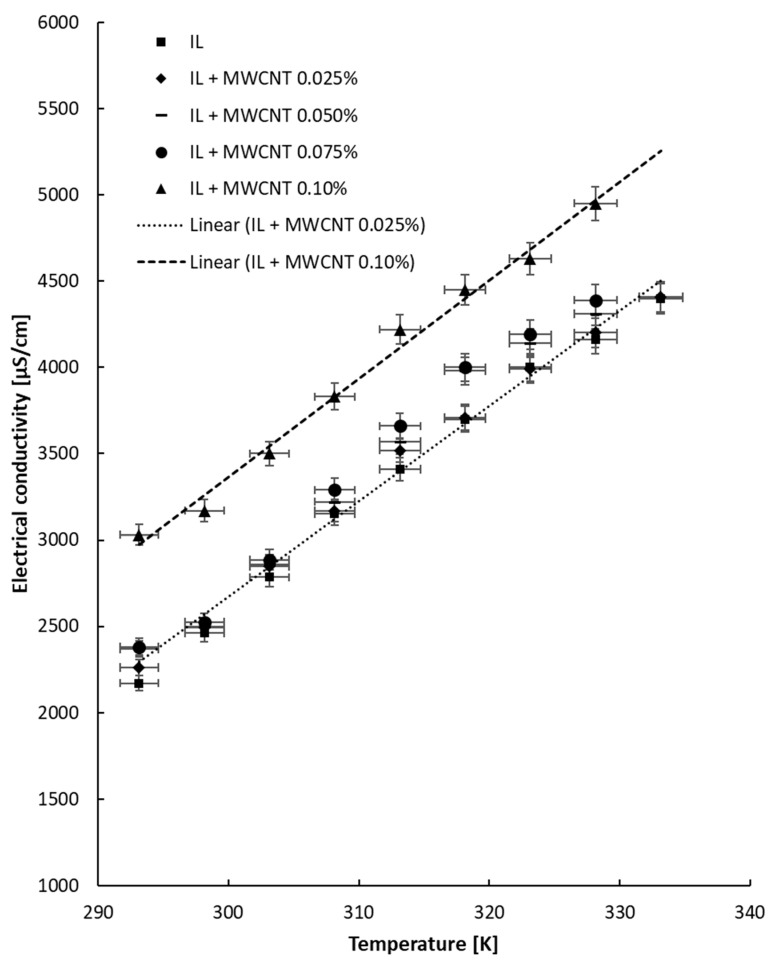
Experimental results for electrical conductivity variation with temperature: MWCNT + IL samples.

The electrical conductivity experiment with heating was performed up to 333.15 K, and the outcomes are given in Figure 4, Figure 5 and Figure 6 separately for each ionanofluid. From the data collected at different temperatures for all samples, it can be noticed that the electrical conductivity linearly increases with temperature. The increase is correlated by means of a linear fit, and the correlation coefficients are listed in Table 4. The equation is as follows:

κ = a T − b (5)

The results are in agreement with the state of the art: the electrical conductivity linearly increases with rise in temperature (see, for example [3,4,5,6,7,8,9]).

Generally, the collected information shows that the electrical conductivity increases with temperature by 63–116% in comparison with the data collected at 293.15 K. The improvement is defined as the ratio between electrical conductivity at 333.15 K and that registered at ambient temperature and clearly depends on the behavior of each suspension during heating. Interestingly, the highest increase was noted for the ionic liquid, while the lowest increase (63%) was observed for the most concentrated suspensions with MWCNTs. Moreover, for alumina- and ZnO-based suspensions, the increase in electrical conductivity is similar for all NP loadings and is situated at around 110% and 100%, respectively. From the experimental observations, we can conclude that the addition of nanoparticles slows the rise in electrical conductivity with temperature, and this can be explained by the solid NP addition inside the ionic liquid; however, more tests are needed for a definitive explanation.

**Figure 7:**
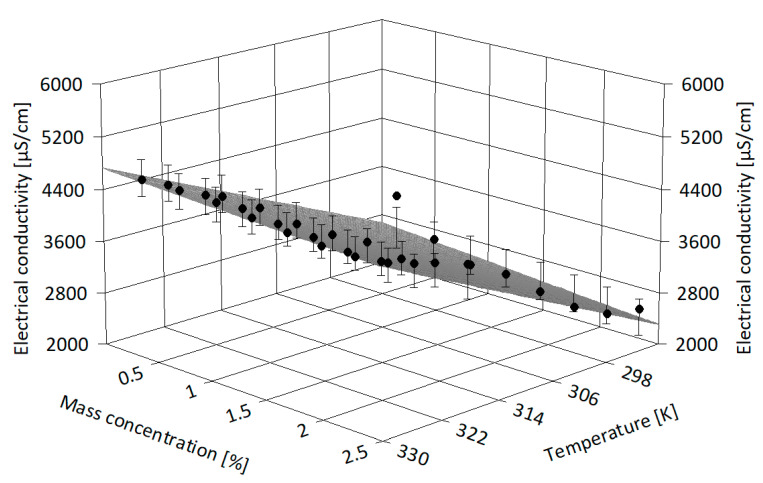
Three-dimensional analysis of alumina suspensions.

**Figure 8:**
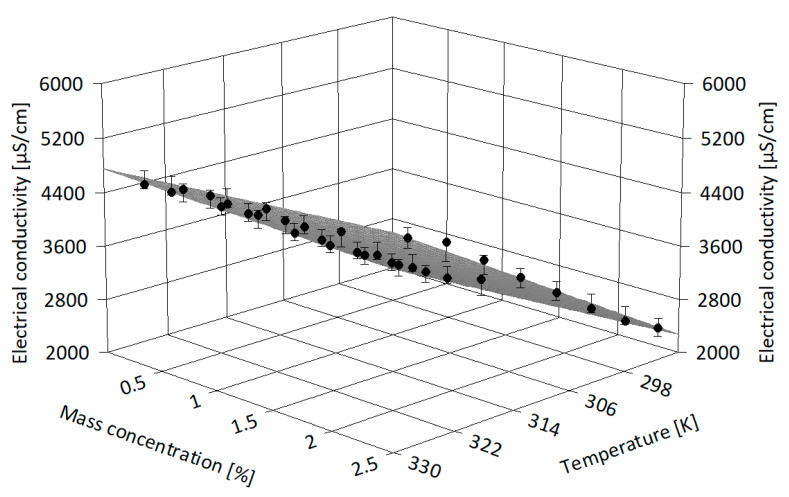
Three-dimensional analysis of ZnO suspensions.

**Figure 9:**
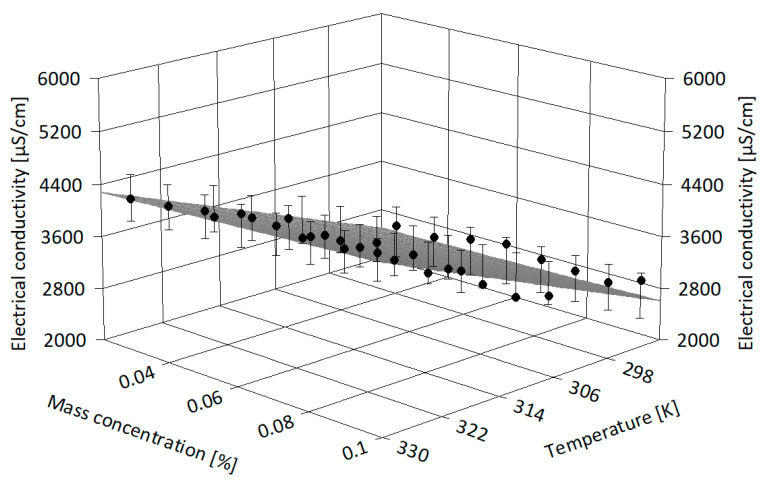
Three-dimensional analysis of MWCNT suspensions.

The experimental data were correlated using Table Curve 3D v4.0.01 software [36] in order to derive more information on electrical conductivity dissimilarity with both NP loading and temperature (see Figure 7, Figure 8 and Figure 9). Figure 7 depicts the alumina-based suspension experimental points and the regression surface, and it is clear that the electrical conductivity moderately decreases with mass concentration and increases with temperature. A similar interpretation can be made from Figure 8 and Figure 9 for samples of IL with ZnO and MWCNT, respectively. The polynomial regressions that are valid for investigated samples (i.e., in terms of nanoparticle type and mass concentration) and up to 333.15 K can be expressed as follows:

-For alumina suspensions:

κ_nf_ = −18425.97 + 70.17 T + 155.22 ϕ (6)

-For ZnO suspensions:

κ_nf_ = −18076.97 + 69.18 T + 116.04 ϕ (7)

-For MWCNT suspensions:

κ_nf_ = −15395.54 + 59.10 T + 8667.00 ϕ (8)

where ϕ is the mass concentration and T refers to temperature, in K. Statistical data regarding 3D correlations are given in Table 5.

In Table 5, the adjusted R-squared value accounts for the number of terms in the model, while all the statistical parameters are automatically calculated by the analysis software (see [36] for details). From Table 5, it can be seen that all the proposed regression surfaces have good R-square values and the data fit well, so the proposed 3D correlations give adequate results in the considered ranges of temperature and mass concentration.

### 4.3. Comparison with Analytical Models

**Figure 10:**
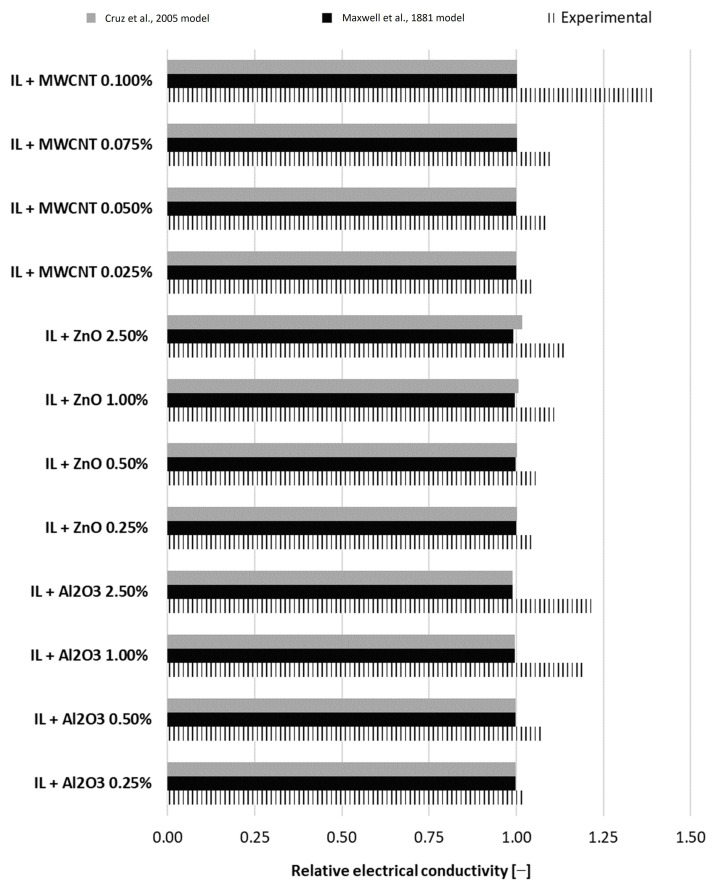
Experimental outcomes versus theoretical models [10,19].

Electrical conductivity also benefits from several theoretical or experimental models, as outlined in Section 2. Nevertheless, the experimental models (see Table 1) are limited to the specific conditions from each experimental situation, such as suspension type, nanoparticle loading, base fluid, etc. Here, a comparison with two theoretical models is made, as can be seen in Figure 10. From the data presented in Figure 10, one can notice that the theoretical models under-predict the experimental data, as was also noted in the literature (see, for example [2,3,30]). This under-prediction is based on the complicated processes that may appear and are not taken into account when theoretical models are applied. On the other hand, Cruz et al.’s model is simple and gives similar values regardless of the nanoparticle type. Other influences may appear due to the synergy between the ionic liquid and nanoparticles, as well as their dimensions.

To conclude, these authors performed an analysis to determine if the discussed suspensions’ electrical conductivity can be estimated with theoretical models, and no agreement was found (see comments and Figure 10). This proves once again that for the electrical conductivity (as well as for other thermophysical properties) of nanofluids, most of the theoretical models underestimate the experimental values. Thus, there is no relevant theoretical approach in discussing the electrical conductivity of a nanocolloid, and experimental investigations remain important when nanocolloid properties are to be estimated.

## 5. Conclusions

In this study, three new nanoparticle-enhanced ionic liquids were prepared, based on [C4mim][BF4] ionic liquid and three kinds of nanoparticles (i.e., Al_2_O_3_, ZnO, MWCNT) with different electrical properties. During this experimental work, the electrical conductivity was thoroughly investigated at ambient temperature and heating up to 333.15 K. The main conclusions of this experimental research are drawn from the results and can be summarized as follows:

- The electrical conductivity of the ionic liquid and the three suspensions with different types of nanoparticles (i.e., an insulator, a semiconductor and a conductor) was carefully investigated at ambient temperature and between 293.15 and 333.15 K. The results were discussed considering the state of the art.
- The test performed at ambient temperature revealed that all suspensions have a higher electrical conductivity compared to the host fluid, and the increase depends on nanoparticle type and nanoparticle loading. The relative electrical conductivity dependence on nanoparticle mass addition is a second-order polynomial one and not linear, as was described in the literature for nanofluids based on water or ethylene glycol.
- The tests performed with heating revealed that the electrical conductivity linearly increases with temperature, as was also demonstrated in the literature.
- All the experimental data were correlated in terms of nanoparticle concentration in the base fluid and temperature influence, as well as in terms of 3D correlations (i.e., based on both mass concentration and temperature influence).
- All the experimental data were compared with two theoretical models and no correspondence was found to be acceptable.

In conclusion, even if this research clarifies several aspects of ionic liquids’ behavior, larger experimental tests are needed for a full picture of the conduct of ionic liquids and nanoparticle-enhanced ionic liquids in real-world applications.

Future research is needed to clarify the electrical behavior of nanocolloids, especially those based on ionic liquids due to their applications at higher temperatures. Moreover, different nanoparticle loadings need to be investigated to confirm the possible modification of electrical behavior at a certain value. Additionally, the studies on electrical behavior must be expanded to dielectric behavior as well as dielectric breakdown voltage, in agreement with applications in the area of high-voltage systems and proton exchange membrane fuel cells.

## Figures and Tables

**Table 1:** Models for electrical conductivity.

Model Type	Model	Equation	Observations
Theoretical	Maxwell model [19]	$\frac{\kappa_{\mathrm{nf}}}{\kappa_{\mathrm{bf}}}=1+\frac{3\left(\left(\frac{\kappa_{\mathrm{np}}}{\kappa_{\mathrm{bf}}}\right)-1\right)\phi }{\left(\frac{\kappa_{\mathrm{np}}}{\kappa_{\mathrm{bf}}}\right)+2-\left(\left(\frac{\kappa_{\mathrm{np}}}{\kappa_{\mathrm{bf}}}\right)-1\right)\phi }$	Maxwell model gives the electrical conductivity as a function of electrical conductivity of nanoparticles (κ_np_) and of the base fluid (κ_bf_), also taking into account the particle volume fraction (ϕ), and is valid for low-volume concentrations of NPs.
	Cruz et al. [10]	(i) $\frac{\kappa_{\mathrm{nf}}}{\kappa_{\mathrm{bf}}}=1-\frac{3}{2}\phi$, for κ_np_ « κ_f_ (insulating NP) (ii) $\frac{\kappa_{\mathrm{nf}}}{\kappa_{\mathrm{bf}}}=1$, for κ_np_ = κ_bf_ (equal conductivity) (iii) $\frac{\kappa_{\mathrm{nf}}}{\kappa_{\mathrm{bf}}}=1+3\phi$, for κ_np_ » κ_bf_ (high conducting NP) κ_nf_/κ_bf_ is the relative conductivity	The three equations demonstrate the theoretical effect, according to Maxwell’s model, of the particle volume fraction on the relative conductivity for a constant value of relative conductivity.
	Bruggeman [32]	$1-\phi =\frac{\kappa_{\mathrm{np}}-\kappa_{\mathrm{nf}}}{\kappa_{\mathrm{np}}-\kappa_{\mathrm{bf}}}{\left(\frac{\kappa_{\mathrm{bf}}}{\kappa_{\mathrm{nf}}}\right)}^{1/3}$	
Experimental	Chereches and Minea [9]	-for silica nanocolloids: κ_nf_ = 354.57 φ − 16.57 -for titania nanocolloids: κ_nf_ = 388.11 φ + 337.29	Valid for silica and titania nanofluids dispersed in water.
	Ganguly et al. [5]	$\frac{\kappa_{\mathrm{nf}}-\kappa_{\mathrm{bf}}}{\kappa_{\mathrm{bf}}}=3679.049\phi +1.085799T-43.4384$	Linear relation was noticed between electrical conductivity and NP volume fraction. Correlation is valid for alumina nanofluids.
	Glover et al. [11]	no correlation proposed, even if a linear increase of electrical conductivity was plotted against mass concentration of carbon nanotubes	Linear relation was noticed between electrical conductivity and NP mass concentration. The experiment was performed for single-wall carbon nanotubes in aqueous fluids.

**Table 2:** Properties of nanoparticles according to the manufacturer.

Chemical Formula	Al_2_O_3_	ZnO	MWCNT
CAS number	1344-28-1	1314-13-2	308068-56-6
Dimensions	~50 nm	~100 nm	50–90 nm
NP shape	spherical		
Melting point	2313.15 K	2247 k	3925–3970 K
Molecular mass	101.96 g/mol	81.39 g/mol	
Density	4.00 g/cm^3^	5.6 g/cm^3^	2.1 g/cm^3^
Refraction index		2.0041	
Specific heat	773 kJ/kg K at 298.15 K	40.26 J/mol K at 298.15 K	0.7333 kJ/kg K at 318.15 K
Expansion coefficient		4.0 × 10^−6^ K^−1^	
Specific surface	>40 m^2^/g (BET)		28 m^2^/g

**Table 3:** Electrical conductivity of nanoparticles [6,12,14,15].

Nanoparticle Type	Electrical Conductivity, µS/cm	Nanoparticle Behavior	References
alumina	10^−8^	insulator	[6]
ZnO	0.7267	semiconductor	[12,14]
MWCNT	10^9^	conductor	[15]

**Table 4:** Correlation coefficients for each sample according to Equation (5).

Sample	a	b	R-Squared Value
IL	66.29	17,272.49	0.988
IL + Al2O3 0.25%	68.25	17,824.36	0.996
IL + Al2O3 0.50%	67.93	17,668.84	0.996
IL + Al2O3 1.00%	65.84	16,867.30	0.989
IL + Al2O3 2.50%	78.19	20,542.03	0.961
IL + ZnO 0.25%	63.30	16,242.40	0.985
IL + ZnO 0.50%	63.89	16,403.65	0.986
IL + ZnO 1.00%	64.90	16,657.14	0.989
IL + ZnO 2.50%	68.50	17,600.08	0.987
IL + MWCNT 0.025%	57.77	14,577.58	0.992
IL + MWCNT 0.050%	60.74	15,500.53	0.985
IL + MWCNT 0.075%	62.11	15,879.15	0.986
IL + MWCNT 0.10%	57.09	13,762.73	0.991

**Table 5:** Statistical data on polynomial regressions.

Samples	R-Squared Value	Adjusted R-Squared Value	Standard Error of the Regression	F-Value
Al_2_O_3_ + ionic liquid	0.978	0.976	127.928	649.729
ZnO + ionic liquid	0.994	0.993	62.752	2593.595
MWCNT + ionic liquid	0.958	0.953	159.357	325.811

## Data Availability

Data can be made available on request.

## Nomenclature

T	temperature, K
R^2^	accuracy of linear correlations
κ	electrical conductivity of the nanofluid, µS/cm
φ	particle volume fraction
ϕ	particle mass concentration, wt%

## Subscripts

bf	refers to base fluid
nf	refers to nanofluid
np	refers to nanoparticles
r	refers to relative

## Abbreviations

NP	nanoparticle
BF	base fluid
MWCNT	multi-walled carbon nanotubes
NF	nanofluid
EDL	electrical double layer
